# Humoral immune response against two surface antigens of *Chlamydia pecorum* in vaccinated and naturally infected sheep

**DOI:** 10.1371/journal.pone.0188370

**Published:** 2017-11-30

**Authors:** Sankhya Bommana, Evelyn Walker, Marion Desclozeaux, Peter Timms, Adam Polkinghorne

**Affiliations:** 1 Centre for Animal Health Innovation, University of the Sunshine Coast, Sippy Downs, Australia; 2 Central West Local Land Services, Dubbo, Australia; University of the Pacific, UNITED STATES

## Abstract

*Chlamydia pecorum* is a globally recognised livestock pathogen due to the significant clinical and economic impact it poses to livestock producers. Routine serological diagnosis is through a complement fixation test (CFT), which is often criticised for cross-reactivity, poor sensitivity and specificity. Although serology remains the preferred method in veterinary diagnostic laboratories, serological assays based on surface antigens of *C*. *pecorum* have not been established until now. In this study, we evaluated the use of two chlamydial recombinant protein antigens (PmpG and MOMP-G) by a direct IgG ELISA method for detection of ovine anti-chlamydial antibodies. Using the Pepscan method we then identified B cell epitopes across PmpG and MOMP-G proteins, in lambs with (a) naturally occurring asymptomatic *C*. *pecorum* infections (b) *C*. *pecorum*-associated polyarthritis and (c) recombinant PmpG and MOMP-G vaccine. Plasma IgG antibodies to PmpG in natural infection of lambs were detected earlier in infection than CFT and served as an acute phase marker. Antibodies to MOMP-G IgG were significantly heightened in lambs with *C*. *pecorum*-associated polyarthritis. PmpG and MOMP-G specific B-cell epitope mapping revealed epitope responses in immunised lambs cluster with some of the epitope responses in naturally infected lambs. B-cell epitope mapping further revealed that lambs with polyarthritis recognised several unique PmpG (50% frequency, peptide 8, 25, 40, 41 and 50) and MOMP (50% frequency, peptide 50) epitopes in comparison to asymptomatic infections. The findings of this study will have implications towards improved serodiagnosis of *C*. *pecorum* infections in livestock and inform the downstream development of alternative peptide-based antigens for future *C*. *pecorum* vaccine studies.

## Introduction

*Chlamydia pecorum* is an obligate intracellular pathogen and the causative agent of kerato-conjunctivitis [1] and polyarthritis [2] in sheep, sporadic bovine encephalomyelitis (SBE) [3] in cattle and sporadic abortions in goats [4]. Importantly, asymptomatic gastrointestinal tract infections are common in livestock and are linked to subclinical pathological effects in dairy cattle [5, 6]. In Australia, *C*. *pecorum* is a highly prevalent asymptomatic infection in sheep with longitudinal analysis revealing that more than 30% of the nation’s sheep shed this pathogen [7]. While the exact costs incurred due to these infections are unclear, the economic impact of bacterial arthritis in lambs, of which *C*. *pecorum* is one of the major causes, is estimated to be >$30M per year [8]. Of note, this same pathogen also poses a serious threat to the declining population of the iconic marsupial species, the koala (*Phascolarctos cinereus*) [9], with spill-over from infected livestock suspected to be one of the sources [10, 11].

In sheep, *C*. *pecorum* associated polyarthritis is difficult to diagnose due to a lack of clarity over clinical presentation [2], particularly since multiple aetiological agents (eg. *Erysipelothrix rhusiopathiae* (*E*. *rhusiopathiae*), *Histophilus somni* (*Hemophilus somnus*) *Mycoplasma* spp.) can also cause arthritis in sheep [12]. To further add to this problem, routine serological diagnosis is through a complement fixation test (CFT) which relies on detection of antibodies to the chlamydial genus-specific lipopolysaccharide antigen or whole *Chlamydia* elementary bodies [13], leading to inevitable serological cross-reactivity (poor specificity) between *Chlamydia* species, *Chlamydia*-related organisms and other gram-negative bacteria (eg. *Escherichia coli*, *Salmonella* spp. and *Acinetobacter* spp.) [14, 15]. CFT also has variable sensitivity depending on the host species and antibody isotype [16, 17], emphasising the need for alternative assays to improve the diagnosis and treatment of *C*. *pecorum* infected animals.

For other ovine pathogens, such as *C*. *abortus*, these efforts are already underway and have focussed on the identification of novel recombinant antigen proteins that may serve as the targets for species-specific ELISA assays [18]. This strategy holds some promise although the cross-reactivity observed for a number of the analysed proteins to those from other chlamydial species was a significant limiting factor [18]. To overcome some of these issues, others have utilised in-silico prediction methods [19] and screening synthetic peptide libraries in order to capture antibodies that are specific to several chlamydial polymorphic immuno-dominant antigens (OmpA, CT618, PmpD, IncA, CT529, CT442, IncG, Omp2, TarP, and IncE proteins) for each of the currently described chlamydial species [19]. This work has revealed some promising new antigens for development of a *C*. *pecorum* species-specific ELISA for cattle but no validation studies have yet been done for *C*. *pecorum* infected sheep and koala.

Beyond the urgent need for *C*. *pecorum* species-specific serology tests for the diagnosis of infections in sheep, little is still known about the immune response of sheep to this enigmatic pathogen. The latter is particularly problematic if alternative vaccine-based control strategies are to be developed for *C*. *pecorum*. We recently trialled a prototype *C*. *pecorum* vaccine, consisting of the chlamydial major outer membrane protein (MOMP-G) and polymorphic membrane protein G (PmpG) in lambs and their pregnant ewes [20]. This antigen selection was based on our ongoing work in *C*. *pecorum* infected koalas that showed that vaccinated animals can make strong and long lasting humoral and cell-mediated immune responses to these antigens that recognised and neutralised whole chlamydial elementary bodies (EBs) [21, 22]. Recombinant MOMP-G and PmpG were also found to be immunogenic in immunised lambs, resulting in the production of specific systemic IgG and secretory IgA responses, however, the vaccine failed to trigger production of neutralising antibodies despite being seemingly able to recognise whole chlamydial EBs [20].

To investigate the immune response of *C*. *pecorum* infected lambs with further efforts to improve the diagnosis, treatment and control of this pathogen, we evaluated the use of two chlamydial recombinant protein antigens (MOMP-G and PmpG) by direct IgG ELISA for the detection of ovine anti-*C*. *pecorum* antibodies. These assays were then used to compare the humoral immune responses of sheep with different *C*. *pecorum* infection and disease outcomes, to provide baseline data to inform the development of improved species-specific sero-diagnostics and an improved epitope-based *C*. *pecorum* vaccine strategy.

## Results

### Comparison of recombinant protein antigen ELISAs versus the chlamydial CFT

**Fig 1:**
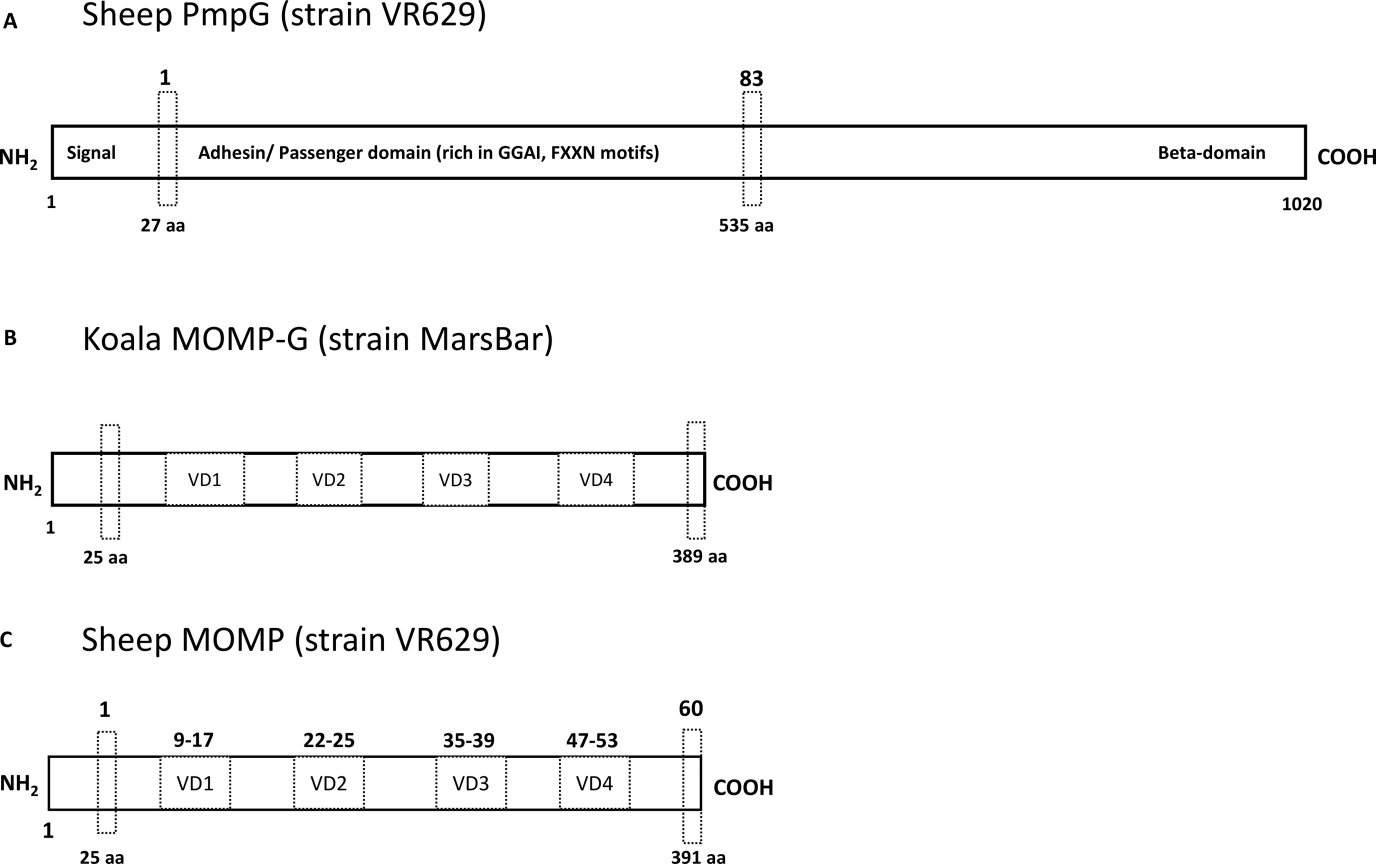
Diagrammatic representation of the recombinant antigens and peptides used in the direct IgG ELISA and peptide ELISA formats. Recombinant PmpG and peptide 1 to 83 represent the adhesin domain (amino acid 27 to 535) rich in GGAI and FxxN motifs (A), recombinant MOMP-G represents the near full-length region (amino acid 25 to 389) of koala MOMP-G MarsBar strain consisting of conserved and variable domains 1, 2, 3 and 4 (B), and peptide 1 to 60 represent the near full-length region (amino acid 25 to 391) of sheep MOMP VR629 strain consisting of conserved and variable domains (C).

In a first step to evaluating the immune response of *C*. *pecorum* infected sheep, we compared the diagnostic performance of in-house antigen-specific anti-PmpG and anti-MOMP-G IgG ELISAs (Fig 1) to the commercially available CFT that detects antibodies to whole chlamydial elementary bodies, in different groups of sheep with *C*. *pecorum* infections. We tested a total of 86 samples, comprising 66 plasma samples collected as part of a previous study of *C*. *pecorum* infections on a commercial sheep farm (S1–S3 Tables) and 20 serum samples of lambs with overt signs of polyarthritis from several flocks across NSW, Australia (Table 1).

**Table 1:** Description of origin, *C*. *pecorum* status and sampling characteristics of samples used in the study.

Group	No. of lambs	No. of samples	Sample characteristics
Clearance of infection	13	39	Lambs from a commercial farm in NSW, Australia with *C*. *pecorum* infections; positive by PCR and CFT. Samples were tested at 2, 6 and 10 months of age.
Recurring infections	7	21	Lambs from a commercial farm in NSW, Australia with repeated *C*. *pecorum* infections; positive by PCR and CFT. Samples were tested at 2, 6 and 10 months of age.
Recurring infections	3	6	Lambs from a commercial farm in NSW, Australia with *C*. *pecorum* infections; repeatedly positive by PCR at more than one time point and negative by CFT during 2 to 10 months.
Polyarthritis	20	20	Sheep from several flocks in NSW, Australia with clinical evidence of polyarthritis and *C*. *pecorum* positive by CFT (Cross-sectional sampling).
Total	43	86	
Vaccinated lambs	4	4	Lambs from the vaccine safety trial received prototype *C*. *pecorum* vaccine at the time of birth and 12 weeks post-birth. Samples were tested at 24 weeks post-birth.
Negative control groups	16	16	Uninfected lambs (n = 12) from a commercial farm in NSW, Australia, *C*. *pecorum* PCR and CFT negative, these animals are from the 2 month sampling time point of “clearance of infection” group (see above). Pre-immunized lambs (n = 4) from the vaccine safety trial served as negative controls for epitope mapping experiments of vaccinated lambs.

In these samples, the overall CFT positivity was 55.8% (48/86; Table 2). The same panel of sera had positivity of 77.9% (67/86) for the individual anti-PmpG and anti-MOMP-G IgG ELISAs (Table 2). The positive and negative percentage agreement for each of these assays was then compared individually to the CFT result (Table 3). For the anti-MOMP-G IgG ELISA, the positive percentage agreement (PPA) was 91.7%, including 44/48 samples that were positive by both assays (Table 3). The negative percentage agreement (NPA) of 42.1%, was much lower, however, with only 16 samples negative by both assays and 22 positive by anti-MOMP- IgG ELISA but negative by the CFT (Table 3). The anti-PmpG IgG ELISA revealed similar results with a PPA of 97.9% (47/48) and NPA of 47.36% with only 18 samples negative by both assays and 20 positive by PmpG IgG ELISA but negative by CFT (Table 3).

**Table 2:** Comparison of CFT, anti-MOMP-G and anti-PmpG IgG ELISA positivity in lambs with naturally occurring *C*. *pecorum* infection groups (a) clearance of infection (n = 39) (b) recurring infections (n = 27) and (c) polyarthritis (n = 20). Statistical significance was determined by two-tailed Fisher’s exact test by analysing a 2x2 contingency table for (a) CFT vs. PmpG and (b) CFT vs. MOMP-G.

	Clearance of infection			Recurring infections			Polyarthritis			Overall (n = 86)		
	CFT	MOMP	PmpG	CFT	MOMP	PmpG	CFT	MOMP	PmpG	CFT	MOMP	PmpG
No of positives	18	28	24	10	19	23	20	20	20	48	67	67
% positive	46.15	71.79	61.54	37.04	70.37	85.19	100	100	100	55.8	77.9	77.9
Fisher's exact test P-value		0.0375	0.256		0.0281	0.0006		-	-		0.0019	0.0019

**Table 3:** Characteristics of CFT and ELISA positivity, positive and negative percentage agreement (PPA and NPA) of MOMP-G and PmpG IgG ELISA with CFT.

ELISA method	No. of samples tested	CFT+ ELISA+	CFT- ELISA-	CFT- ELISA+	CFT+ ELISA-	PPA	NPA
MOMP-G IgG	86	44	16	22	4	91.66	42.1
PmpG IgG	86	47	18	20	1	97.91	47.36

To investigate the animals that were positive by either PmpG or MOMPG-specific IgG ELISAs but were negative by CFT, where possible, we reviewed the *C*. *pecorum* PCR positivity data for these animals (S1–S3 Tables). In these samples, 68.2% (15/22) of animals that were MOMP-G IgG positive and CFT negative were PCR positive for *C*. *pecorum* (S1–S3 Tables). Similarly, 90.0% (18/20) were positive by PmpG IgG and negative by CFT were PCR positive for *C*. *pecorum* (S1–S3 Tables).

### Humoral immune responses to surface antigens in lambs with naturally occurring *C*. *pecorum* infections

On the basis of the observations of enhanced relative sensitivity for each of the antigen-specific ELISAs, we decided to utilise these assays for further studies to characterise the sheep humoral immune response to *C*. *pecorum* infection. Samples were obtained from a (a) longitudinal cohort of lambs we previously sampled every two months and analysed at 2, 6 and 10 months of age (n = 20); and (b) a serum panel of sheep with clinical signs of polyarthritis and a positive chlamydial CFT (n = 20). Animals in the former cohort were further divided into animals that were *C*. *pecorum* PCR positive at 6 months of age but either (i) became negative at 10 months of age (n = 13; ‘Clearance of infection’) or remained PCR positive at 10 months of age (n = 7; ‘Recurring infections’) (Table 1). The individual details of all animals in these cohorts is provided in S1–S4 Tables.

**Fig 2:**
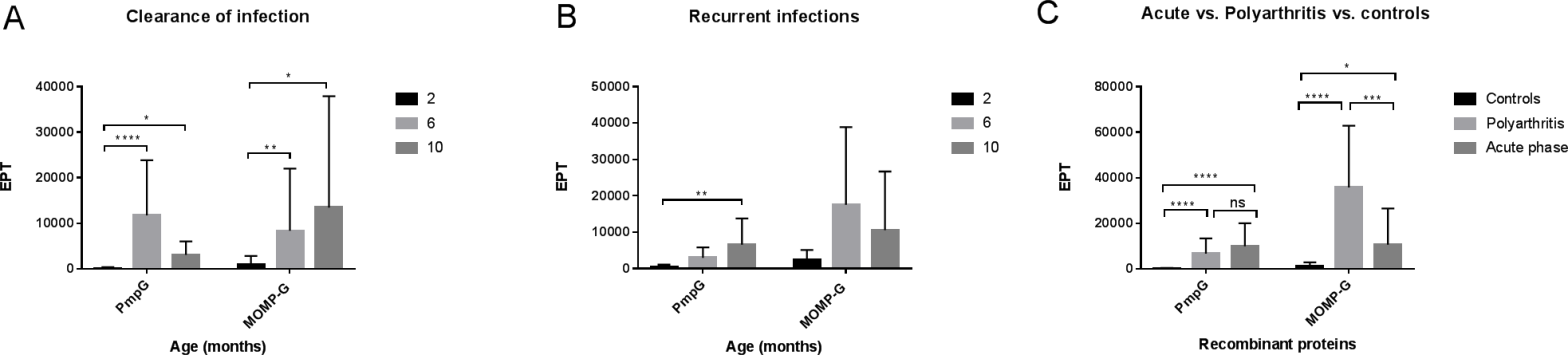
**Anti-PmpG and anti-MOMP-G IgG end point titre (EPT) in lambs with naturally occurring *C*. *pecorum* infections at 2, 6 and 10 months of age, clearance of infection (n = 13) (A), recurring infections (n = 7) (B). Comparison of anti-PmpG and anti-MOMP-G IgG EPT in acute phase infection (n = 33) versus polyarthritis (n = 20) versus negative controls (n = 13) (C).** Fig 2A represents anti-PmpG and anti-MOMP-G IgG EPT values in clearance of infection group, peak infection occurred at 6 months of age followed by infection clearance by 10 months of age. Fig 2B represents EPT values in recurring infections group, predominantly primary infection peak occurred at 6 months followed by a secondary infection at 10 months. Fig 2C represents a cross-sectional comparison of EPT values of (a) acute infections derived from animals at 6 month time point of clearance of infection groups, 2, 6 and 10 month time point of recurring infection groups when lambs were *C*. *pecorum* DNA PCR positive (b) lambs with polyarthritis and (c) negative controls *i*.*e*., lambs at two month time point from clearance of infections group that were *C*. *pecorum* DNA PCR negative).

In animals that cleared their infection, 1/13 (7.7%) animals was positive by PmpG ELISA at 2 months of age despite having no detectable levels of *C*. *pecorum* DNA by PCR. This animal had a low end point titre (EPT) of 1,116 and was positive by CFT (Fig 2, S1 Table). At 6 months of age, the time point when we showed infection levels peak in this cohort (S1 Table), 100% of animals were positive by this assay (13/13) with a peak in PmpG IgG response (Fig 2). The EPT difference was statistically significant compared to the 2 month time point (P<0.0001) (Fig 2). At the final time point of sampling (10 months), PmpG IgG EPTs for 10/13 (84.61%) remained positive (S1 Table), although the mean EPT (3,043) was much lower compared to the 6 month time point (Fig 2). All animals at this latter time point were PCR negative at all sites sampled. The IgG patterns observed by the MOMP-G ELISA revealed a slightly contrasting pattern to that observed in the PmpG IgG ELISA both in terms of overall positivity and EPT trends (Fig 2). Four out of 13 (30.8%) animals were positive for MOMP-G IgG at two months of age with low EPTs (Fig 2). Similar to the PmpG IgG ELISA, at 6 months of age, all animals became positive with a peak in EPT titers (Fig 2). This rise in EPTs at 6 month was statistically significant compared to the 2 month time point (P<0.0001). Interestingly, while the EPTs were clearly variable, at 10 months the EPTs continued to rise even though the animals became PCR negative and this pattern contrasted with the PmpG IgG ELISA results (Fig 2). When this rise in EPTs at 10 month was further assessed, 11/13 (84.61%) animals were positive for MOMP-G IgG, 6/13 (46.15%) animals went on to make higher MOMP-G IgG antibodies than at 6 month and 5/13 (38.46%) antibodies declined and 2/13 (15.38%) had no antibodies detected to this antigen (S1 Table).

In the animals that remained PCR positive at 6 and 10 months (recurring infections), PmpG IgG ELISA titers remained relatively low throughout (Fig 2), gradually rising from 2 to 10 months (P = 0.0023). PmpG IgG positivity rates were similar to that observed in the ‘clearance of infection’ cohort, with 4/7 (57.1%), 7/7 (100%) and 7/7 (100%) animals positive at 2, 6 and 10 months (S2 Table). In the MOMP-G IgG ELISA results, in contrast to the latter assay, MOMP-G IgG titers were higher at 6 months although not statistically significant. While titers continued to rise for PmpG IgG, MOMP-G IgG titers gradually decreased at 10 months of age, however. MOMP-G IgG positivity rates were 3/7 (42.85%), 5/7 (71.42%) and 7/7 (100%) at 2, 6 and 10 months, respectively (S2 Table).

We also performed a cross-sectional analysis in animals with clinical signs of polyarthritis (n = 20) (S3 Table) to compare their PmpG and MOMP-G IgG antibody titers with animals at their acute phase of infection (n = 33) defined as samples from animals at 6 month peak infection time point of ‘Clearance of infection’ group (S1 Table) and *C*. *pecorum* DNA PCR positive at 2, 6 and 10 month time points of ‘recurring infections’ group (S2 Table). A control group, defined as samples from animals collected at the 2 month time point when all animals were PCR negative and 12/13 were CFT negative, was also evaluated (Fig 2, S1 Table). In animals with polyarthritis, 20/20 (100%) were positive for PmpG IgG with a rise in EPTs. This rise in EPTs was statistically significant compared to the control sample titers (P = 0.0001) but almost indistinguishable to the overall positivity (32/33) and EPTs rise recorded for animals with acute infections (Fig 2). The latter PmpG IgG responses were also found to be significantly higher in comparison to control animals (P<0.0001).

Examining the same cohorts with the anti-MOMP-G IgG ELISA revealed a contrasting result. Statistically significant differences were observed in MOMP-G IgG EPTs at acute phase versus polyarthritis lambs (P = 0.006) and polyarthritis versus controls lambs (P<0.0001) (Fig 2), indicating MOMP IgG as a potential marker for polyarthritis due to the heightened IgG response observed in polyarthritis lambs (Fig 2).

### PmpG and MOMP-G B-cell epitope response in lambs with natural infections

On the basis that recombinant *C*. *pecorum*- specific PmpG and MOMP-G IgG could be recognised by naturally infected sheep with different infection outcomes, we were interested in further characterising the antigen-specific epitopes recognised in these animals using a library of 60 MOMP-G and 83 PmpG peptides (Fig 1). To establish a baseline response profile for analysis of naturally infected animals, plasma from four healthy *C*. *pecorum* PCR and CFT negative lambs were evaluated. The mean plus two times standard deviation (Mean+2*SD) of optical density (OD) values for individual peptides were used to subtract away from the OD values to individual peptides in our experimental animals (S5 Table).

**Fig 3:**
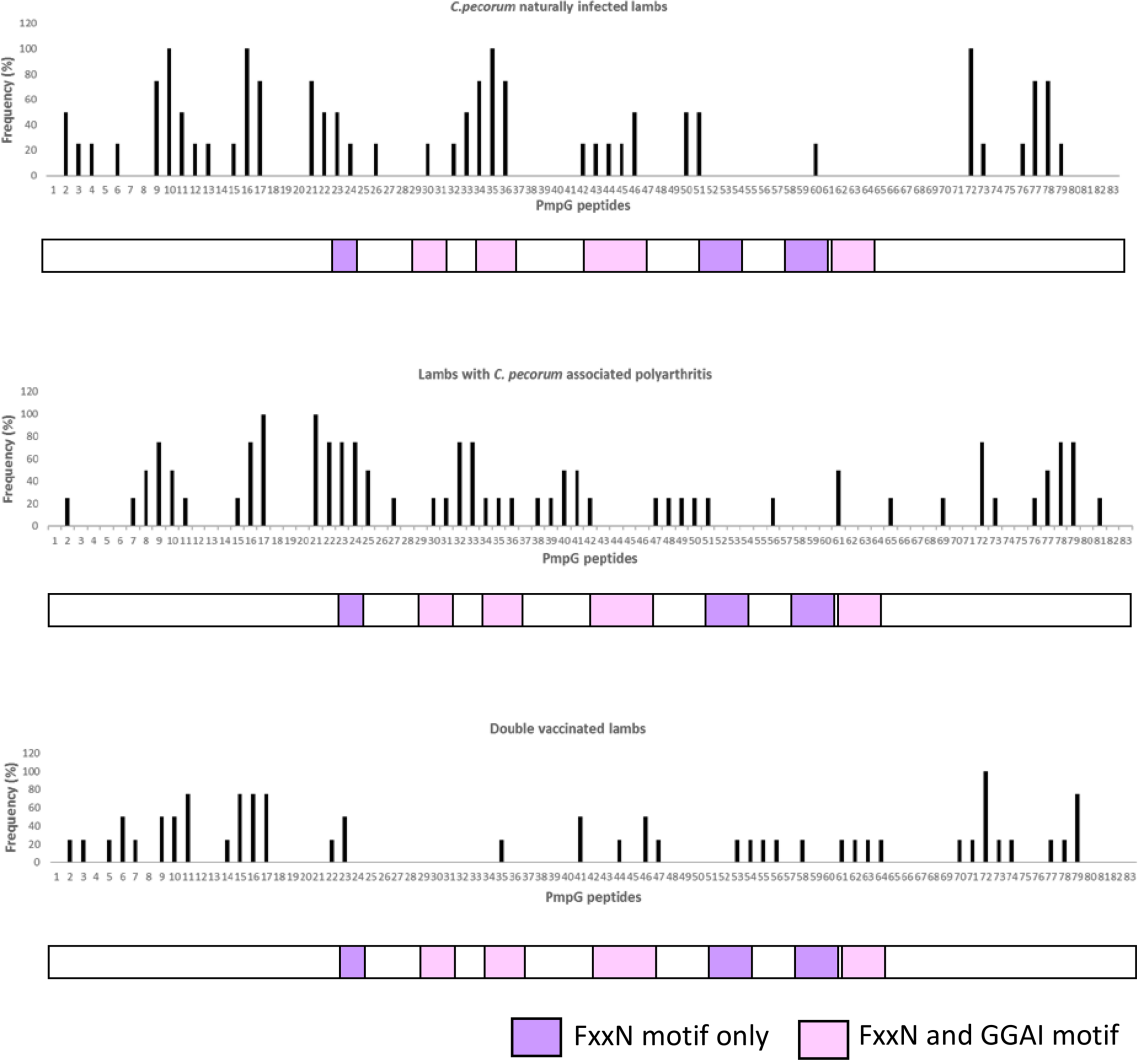
**PmpG B cell epitope mapping in *C*. *pecorum* naturally infected lambs (n = 4) (A), lambs with *C*. *pecorum-* associated polyarthritis (n = 4) (B), and *C*. *pecorum* PmpG vaccinated lambs (n = 4) (C).** The bars indicate frequency (%) of lambs that responded to the corresponding peptide. Below each graph is a linear map of PmpG protein; purple boxes represents FxxN motifs only and pink boxes represent the region rich in FxxN and GGAI motifs.

Fig 3 represents the PmpG epitope response profiles of asymptomatically infected animals and animals with polyarthritis. Overall, the profiles in both these groups exhibited strong immunological recognition in the adhesion region rich in FxxN and GGAI motifs (50–100% frequency, peptide 21–24 and 33–36) and also outside of this region (25–100% frequency, peptide 8–11, 16, 17, 72, 78, 79) (Fig 3). In the four asymptomatically infected lambs, particularly, strong responses were recorded in the FxxN and GGAI motifs (75–100% frequency, peptides 34–36) and regions outside (75–100% frequency, peptides 9, 10, 16, 17, 21, 72, 77, 78). The PmpG epitope response profile in four lambs with polyarthritis was somewhat similar with high epitope response in FxxN and GGAI motifs (75–100% frequency, peptides 22–24, 32, 33) and regions outside (75–100% frequency, peptides 9, 16, 17, 21, 72, 78, 79) (Fig 3). In terms of unique epitopes for each cohort, epitopes exclusively recognised by animals with naturally occurring infections were in the FxxN and GGAI motifs (25–50% frequency, peptides 43–46 and 60) and regions outside (25% frequency 3, 4, 6, 12, 13, 26) (Fig 3). Epitopes unique to the polyarthritis groups were identified predominately in regions outside of functional motifs (25–50% frequency, peptide 7, 8, 25, 27, 31, 38, 39, 40, 41, 47, 49, 56, 65, 69 and 81) and one epitope in the FxxN and GGAI motif (Peptide 61) presented with 50% frequency (Fig 3).

**Fig 4:**
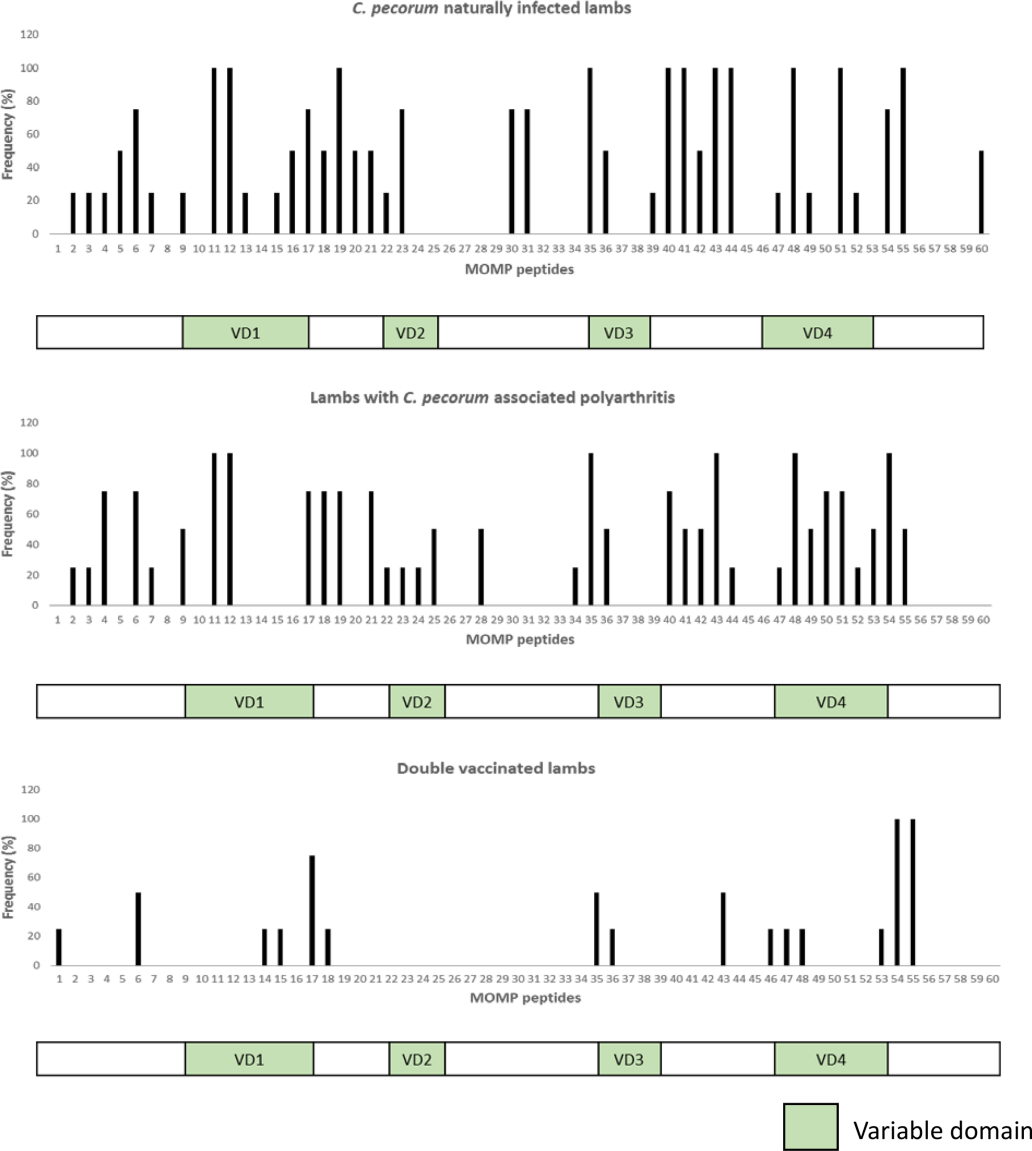
**MOMP B cell epitope mapping in *C*. *pecorum* naturally infected lambs (n = 4) (A), lambs with *C*. *pecorum-* associated polyarthritis (n = 4) (B), and *C*. *pecorum* MOMP-G vaccinated lambs (n = 4) (C).** The bars indicate frequency (%) of lambs that responded to the corresponding peptide. Below each graph is a linear map of MOMP protein; VD represents the variable domain.

Fig 4 illustrates the MOMP-G response profile of asymptomatically infected lambs. The epitope response profiles in this group indicate strong responses in the VD1 (100% frequency, peptide 11,12), VD2 (75% frequency, peptide 23), VD3 (100% frequency, peptide 35), VD4 (100% frequency, peptide 48, 51, 54, 55) and conserved domains (75–100% frequency, peptide 19, 40–44) (Fig 4). Analysis of the lambs with polyarthritis (Fig 4) revealed a somewhat similar response to lambs with naturally occurring infections (Fig 4) with strong responses to VD1 (100% frequency, peptide 11,12), VD3 (100% frequency, peptide 35) and VD4 (100% frequency, peptide 48, 54, 55) and conserved domains (75–100% frequency, peptide 17–19, 21 and 41–43). The overall number of peptides recognised was higher in the asymptomatically infected animals than the polyarthritis cohort, however, the overally frequency of recognition of these unique epitopes in this cohort was relatively low (≤ 50%). Only one peptide (peptide 50) was unique to the polyarthritis group with a frequency ≥ 75% (Fig 4).

### Some PmpG and MOMP-G peptides recognised in animals with natural *C*. *pecorum* infection are also recognised in animals immunised with the recombinant PmpG and MOMP-G proteins

To study the humoral response of vaccinated lambs in more detail and to compare the immune response of these animals to sheep with naturally occurring *C*. *pecorum* infections, epitope mapping was performed on the plasma of four healthy pre-immunised and post-immunised *Chlamydia* negative lambs (Figs 3 and 4). Pre-immunisation sample results were used to establish a background for analysis of post-immunisation samples (S5 Table).

For PmpG, a larger number of peptides (36/83; 43.3%) were recognised in the immunised animals relative to the naturally infected animals (37/83; 44.57%), although the individual response of animals was again highly variable (Fig 3). Six peptides from the conserved regions of PmpG protein were recognised with ≥75% frequency in immunised lambs (11, 15, 16, 17, 72, and 79) and were also presented with ≥75% frequency in naturally infected lambs, suggestive of a potentially immunodominant region in the *C*. *pecorum* PmpG protein (Fig 3). Peptide 33–36 is a region of PmpG rich in FxxN and GGAI motifs that was not recognised in the immunised sheep sera but could be detected in naturally infected animals (Fig 3).

Fig 4 represents the MOMP-G vaccine-induced response profile of post-immunisation plasma of four lambs that were *Chlamydia* negative and received the prototype *C*. *pecorum* vaccine. Again, the response of individual animals was highly variable with significantly less epitopes (15/60; 25%) recognised in the vaccinated animals compared to naturally infected animals (37/60; 61.66%) (Fig 4). Of these, only three peptides from the VD1 and conserved domain (17, 54 and 55) displayed a recognition frequency of >75% and were also recognised with ≥75% frequency in the naturally infected animals suggesting that these epitopes are highly immunodominant in MOMP (Fig 4).

## Discussion

We have investigated the profile and development of the humoral immune response to two surface-exposed *C*. *pecorum* proteins, PmpG and MOMP-G, during *C*. *pecorum* infection in sheep that experienced different infection (clearance of infection and recurring infections) and disease outcomes (*C*. *pecorum* induced asymptomatic infections and polyarthritis). In doing so, we showed that our newly developed recombinant ELISAs to each recombinant protein performed better than the existing chlamydial CFT. When we compared the serological responses to each of these proteins in more detail, we found evidence of distinct antibody response patterns for each of these proteins that may be of diagnostic value, with high titre antibody responses to PmpG observed during active stages of infection and strong antibody response to MOMP-G observed in sheep with polyarthritis. Epitope mapping of these proteins was then performed. While considerable variability could be observed in the individual responses of animals in each cohort studied, we were nevertheless able to identify a short-list of peptide-derived epitopes for each protein that were recognised by both naturally infected animals and animals vaccinated with each of the recombinant proteins. Together, these experiments are anticipated to pave the way for evaluation and validation of protein and peptide-based *C*. *pecorum*-species specific ELISAs as well as providing a short-list of potential peptide antigen candidates to form the next phase of *C*. *pecorum* vaccine development in sheep.

The complement fixation test (CFT) is the only test currently recognized by the World Organization for Animal Health (OIE) and the Sub-Committee for Animal Health Laboratory Standards (SCAHLS) for diagnosing ovine chlamydiosis [13, 23]. In this study, we evaluated the diagnostic potential of two antigens, PmpG and MOMP-G, in the development of sero-diagnostic assays for *C*. *pecorum*. Based on our findings, we found that the PmpG ELISA IgG response is highly specific for *C*. *pecorum* infection, since none of the healthy *C*. *pecorum* PCR and CFT negative lambs revealed any antibodies against PmpG in their sera. Interestingly, PmpG IgG responses could be detected earlier in infection than CFT, suggesting its potential utility as an acute phase marker of infection. This observation is consistent with a recent *C*. *abortus* serology-based study that found Pmp13G responses were highly specific and detected early in *C*. *abortus* infections [18].

In contrast, while the MOMP-G IgG response was found to be more readily detected than the CFT response, the detection of MOMP-G antibodies in 4/12 healthy *C*. *pecorum* PCR and CFT negative lambs, raises questions over the specificity of this response. It is conceivable that these false positives may be due to maternal antibodies from ewes since these were detected in two months old lambs or to an infection early on post-birth (S1 Table). While the PmpG antibody response could be detected earlier in the longitudinal time course of infected animals, MOMP-G IgG antibodies, overall, persisted longer than the anti-PmpG IgGs. This strong MOMP-G IgG response was particularly elevated in sheep with *C*. *pecorum*-associated polyarthritis in comparison to acute asymptomatically infected animals. Similar observations have been made for *C*. *abortus*, where aborting sheep developed a strong antibody response to MOMP along with several other virulence associated proteins such as CPAF, TARP, and the SINC homolog CAB063 [18, 24]. The heightened MOMP-G IgG response observed during *C*. *pecorum* induced polyarthritis in lambs can be linked to studies [25] that suggest MOMP as an important mediator of immunity, chlamydial persistence and disease pathogenesis, in this case being polyarthritis. Although this finding suggests that MOMP is an important marker for polyarthritis, further studies are required to investigate the potential specificity of the anti-MOMP IgG response based on the results of this work but also previous serology studies in *C*. *abortus* and *C*. *trachomatis* [18, 24, 26, 27].

To further understand the variation in immune recognition and specificity of the antibody response, we performed B-cell epitope mapping along the near full-length MOMP and adhesion region of PmpG proteins in (a) lambs with asymptomatic *C*. *pecorum* infections; (b) sheep with *C*. *pecorum*-associated polyarthritis; and (c) recombinant MOMP-G and PmpG vaccinated lambs. When we evaluated sera from four naturally infected lambs, interestingly, 11 MOMP and four PmpG linear epitopes were recognised with 100% frequency in all the four animals tested. Apart from these 11 MOMP and four PmpG immunodominant linear epitopes, 26/60 MOMP and 33/83 PmpG epitopes were recognised with ≤ 100% frequency which means these epitopes were not recognised by all the four lambs. These four lambs belonged to the same Border Leicester flock but were outbred and possibly explains why they recognised a diverse set of epitopes with variable frequencies. Beyond the genetic background of these animals, another possible explanation could be that these animals are exposed to different strains of *C*. *pecorum* circulating in the flock. While we did not have any molecular data on the strains circulating in these flocks, previous work in sheep by our group [10, 11] and by others in cattle [28], have revealed a complex on-farm strain diversity with animals capable of being infected with multiple genetically diverse strains at different anatomical sites or, indeed, even the same site [11]. To support the likelihood of this explanation, our previous B-cell epitope mapping studies also show that different epitopes were recognised in koalas infected with different *C*. *pecorum ompA* genotypes [29].

When we evaluated sera from four *Chlamydia* negative lambs that were immunised with recombinant MOMP-G and PmpG proteins, we observed slightly different epitope response pattern in comparison to the responses seen in naturally infected lambs (Figs 3 and 4). A total of 15 MOMP and 36 PmpG linear epitopes were recognised and only three of these MOMP epitopes (Peptide 1, 14 and 46) and 12 PmpG epitopes (Peptides 5, 14, 53, 54, 55, 58, 62, 63, 64, 70, 71 and 74) were different to the epitopes recognised during a natural live infection (Figs 3 and 4). In summary, PmpG and MOMP-G B-cell epitope response in natural immune response have a broader distribution than in immunised lambs with some epitopes commonly and frequently presented in vaccine induced and natural immune response. Previous studies have highlighted that natural immune response targets usually have much broader targets and distribution [30], however, it is not clear yet whether an effective vaccine will need to improve on the existing natural immune response or mimic the natural immune response when the host is presented with an infection.

**Fig 5:**
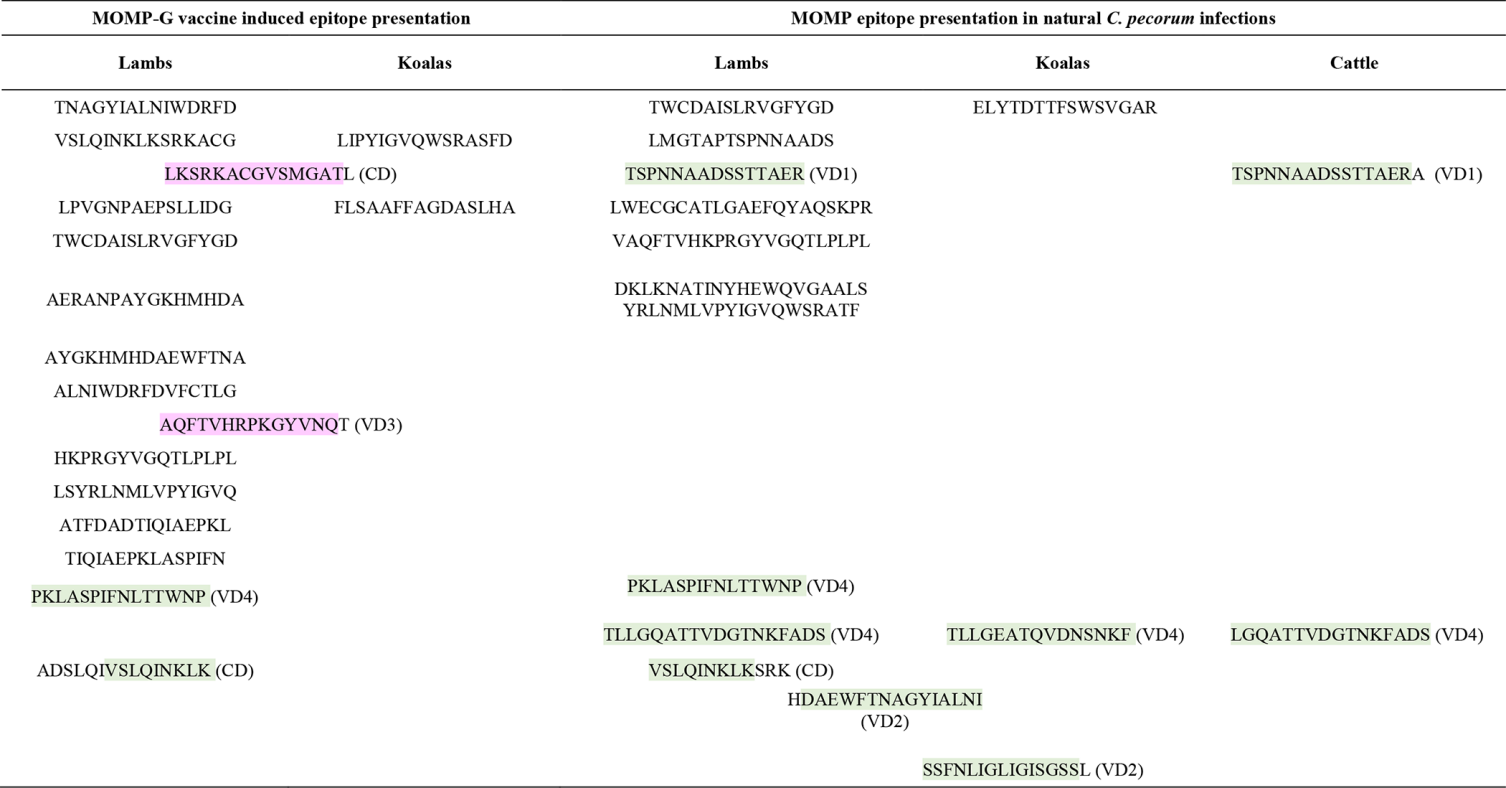
**Comparison of MOMP epitope presentation in (a) vaccinated lambs (present study) versus koalas (Kollipara *et al*., 2013) (b) naturally infected lambs (present study) versus koala (Kollipara *et al*., 2013) versus cattle (Rahman *et al*., 2015).** Peptides highlighted in pink represent epitopes presented in the MOMP protein region that were common to sheep and koala vaccine and green indicate epitopes that were common in naturally infected hosts and in vaccinated lambs.

Our previous koala and sheep vaccine studies also give us the opportunity to identify potential peptide antigens that were recognised independently of the species studied. When the MOMP-G vaccine-induced epitope response in lambs was compared with that of koalas vaccinated with MOMP-G from our previous vaccine trial [29], we found that epitope presentation was more diverse (15 epitopes) in lambs in comparison to koalas (4 epitopes). Two peptides from the conserved domain and variable domains 3 in vaccinated groups, nevertheless, were commonly recognised by both hosts (Fig 5, epitopes highlighted in pink), reinforcing the immunodominant nature of these regions of MOMP. We also found that MOMP epitope responses to naturally occurring *C*. *pecorum* infections in lambs was more diverse than in naturally infected koalas (Fig 5), however, 2/3 epitopes recognised from the variable domains 2 and 4 in koalas were also recognised in lambs (Fig 5, epitopes highlighted in green). The degree of epitope presentation agreement between sheep and koala in this immunogenic region of VD2 and VD4 is interesting considering the fact that MOMP epitope mapping was carried out in four infected lambs and compared with a single koala that was mapped in the previous study (Fig 5). When epitope responses were compared across the three naturally infected hosts of *C*. *pecorum* (sheep vs. koala vs. cattle), the other latter derived from [31] we found that epitopes from the VD2 and VD4 (Fig 5, highlighted in green) were commonly recognised by sheep, koala and cattle, and epitope from VD1 region was commonly recognised by sheep and cattle (Fig 5, highlighted in green). This finding suggests that MOMP epitope recognition in the surface exposed VD2 and VD4 regions is host-independent antigenic hotspot despite the highly variable nature of these domains, meaning it is not the variability itself but the immunodominant nature of these regions, hinting at immune escape.

In conclusion, for the first time, we have characterised humoral responses to surface proteins in naturally occurring *C*. *pecorum* infections in sheep and further looked into the specificity of these responses by B-cell epitope mapping in naturally infected and vaccinated animals. The findings from this study lays the groundwork for (a) screening of chlamydial antigen derived B cell epitopes using the epitope mapping methodology in other immunodominant and virulent- -associated chlamydial antigens and (b) developing peptide based diagnostic assays which aid in screening various healthy versus diseased animals potentially paving the way for *C*. *pecorum* associated polyarthritis specific diagnosis. Similar approaches have already proven to be potentially fruitful in the discovery of new peptide antigens from *C*. *pecorum* infected cattle [31] and in *C*. *trachomatis* infected women [27]. The results of this work and those studies bring the likelihood of developing sensitive and species-specific serology assays for the diagnosis of different chlamydial species tantalisingly close to reality.

## Materials and methods

### Serum samples used in this study

A total of 86 serum samples used in this study were primarily chosen from (a) an existing longitudinal plasma panel derived from animals studied as part of an on-farm investigation into the epidemiology and impact of *C*. *pecorum* infections on prime lamb production (S1–S4 Tables); and (ii) a recently completed *C*. *pecorum* recombinant protein vaccine safety trial [20]. A further serum samples from 20 sheep with overt signs of polyarthritis and tested *C*. *pecorum* CFT positive derived from routine laboratory diagnostic testing services were kindly provided by the Elizabeth MacArthur Agricultural Institute, New South Wales Department of Primary Industries, Menangle, Australia. The collection and testing of these blood samples was approved by the University of the Sunshine Coast Animal Ethics Committee (AN/S/14/31).

Table 1 provides an overview and detailed description of the serum and plasma samples used in this study with further information detailed below.

### Cloning, expression and purification of recombinant proteins (PmpG and MOMP-G)

*C*. *pecorum* (strain VR629) PmpG containing the adhesin domain (amino acid 27 to 520, Fig 1) was expressed and purified as previously described [20]. Similarly, near full-length *C*. *pecorum* (strain MarsBar) MOMP-G protein was also prepared as previously described (amino acid 25 to 389, Fig 1) [32].

### *C*. *pecorum* specific anti-PmpG and anti-MOMP-G IgG ELISA

ELISAs were performed as previously described [20] with minor modifications to the protocol. Briefly, Polysorp plates (Sigma-Aldrich) were coated overnight at 4°C with 2μg/well of His-PmpG, or His-MOMP-G in carbonate bi-carbonate buffer. Blocking was carried out with 200μl/well, 5% skimmed milk powder in 0.01% Tween20- PBS (PBS-T). Following blocking with milk and 3 washes with 0.05% PBS-T, primary antibody, secondary antibody and chromogenic detection steps were carried out at 50μl/well volume. Two-fold serially diluted sera (1:100 starting dilution) was added to the plates and incubated for 1hr at 37°C. Subsequently, after 3 washes with 0.05% PBS-T, HRP-conjugated donkey anti-sheep IgG (1: 10,000, ABCAM) was used as secondary antibody and incubated for 1hr at 37°C. Plates were subjected to washing and TMB substrate (Sigma-Aldrich) was added and incubated for 15 min for chromogenic colour development. The reaction was stopped with 1M H_2_SO_4_ and optical density was read at 450nm. End point titers (EPT) were calculated using non-linear regression (second order polynomial) to determine the point at which the primary sera crosses the negative baseline, cut-off value was identified and antigen-specific antibody end point titre for all samples were generated using GraphPad Prism 7 (GraphPad Software, Inc, La Jolla, CA).

### Design and construction of biotinylated peptide library from *C*. *pecorum* PmpG and MOMP-G sequences

A total of 146 overlapping peptides, each 15 amino acids in length with nine amino acid offset, were designed and screened in this study. Each peptide consisted of a biotin molecule linked to a spacer (-SGSG-) to prevent steric hindrance, followed by the 15mer sequence with an offset of nine amino acids. The PmpG and MOMP sequence based peptides were designed by our group and constructed by Mimotopes (Melbourne, Australia). The adhesin domain of sheep *C*. *pecorum* PmpG (strain VR629) (amino acid 27 to 520, Fig 1) was used to designed 83 overlapping peptides. The near full- length sheep *C*. *pecorum* MOMP (strain VR629) sequence (amino acid 25 to 391) was used to design 60 overlapping peptides (Fig 1).

### PmpG and MOMP-G B cell epitope mapping by biotinylated ELISA

Peptide ELISAs were carried out as follows. Briefly, the lyophilised biotinylated peptides (3-5mg) were reconstituted in 200μl 50% acetonitrile solvent/water mixture. Each peptide was further diluted before use to a working strength of 1:1000 in PBS containing 0.1% BSA and 0.1% sodium azide. High binding streptavidin coated, BSA blocked plates (ThermoFisher Scientific) were coated with 100μl of diluted biotinylated peptides and allowed for the reaction to proceed for one hour at room temperature. 100 μL of the hyperimmune and preimmune sera at a dilution of 1:200 and naturally infected and negative control sera at 1:800 in a dilution buffer of PBS/0.1% Tween 20/0.1% sodium azide was added to each of the wells of the plates containing captured peptides and incubated with agitation for 1 hour at 20°C. The plates were subjected to four washes with PBS/0.1% Tween 20. Bound antibody was detected with a secondary conjugate by adding 100μl of 1;10,000 dilution of HRP-conjugated donkey anti-sheep IgG (ABCAM) in each well and incubated with agitation for 1 hour at 20°C. Following incubation, the plates were washed again for four times with PBS/0.1% Tween 20 and a final wash with PBS only to remove traces of Tween. Peroxidase detection was carried out by adding 100μl of freshly prepared TMB substrate (Sigma-Aldrich) and incubated for 15 min for chromogenic colour development. The reaction was stopped with 1M H_2_SO_4_ and optical density was read at 450nm. Tests were carried out (a) using pre-immune (n = 4) and negative control sera (n = 4) to verify the specificity of the antibody binding observed in experimental animals (b) to check the specificity of the peptides and further reduce the inter-assay variability, the tests on pre-immune and control sera were run in parallel with the hyperimmune and naturally infected sera (c) naturally infected and immunised sample optical density background was calculated from mean plus twice the standard deviation (Mean+2*SD) of negative control and pre-immunised lamb sera, respectively.

### Statistical analysis

Statistical analysis were carried out using GraphPad Prism version 7 (GraphPad Software, LaJolla, CA, USA) and graphical outputs generated by GraphPad Prism and Microsoft Excel 2013. For the CFT and IgG ELISA (PmpG and MOMP-G) comparisons overall and across different groups described in Table 2, we used two-tailed Fisher’s exact test with the p value set at *p<0.05, **p<0.01, ***p<0.005, ****p<0.001. To compare the means of the three matched groups at 2, 6 and 10 month time points (Fig 2) we performed repeated measures Friedman’s One-way ANOVA and non-parametric Dunn’s multiple comparisons test (alpha = 0.05) were carried out with the same p value set as previously stated. The means of unmatched groups (Fig 2) were compared using Kruskal-Wallis One-way ANOVA and non-parametric Dunn’s multiple comparisons test (alpha = 0.05) were performed with set p values mentioned as above. The frequency (%) of epitope response was calculated in Microsoft Excel 2013 for the individual peptides of PmpG and MOMP-G protein, presented in Figs 3 and 4.

## Supporting information

S1 Table Longitudinal data of individual animal tested in clearance of infection group. Characteristics of CFT titre, PCR load, MOMP-G IgG EPT and PmpG IgG EPT results of lambs that cleared their infection by 10 month of age.
(DOCX)

S2 Table Longitudinal data of individual animal tested in recurring infections group. Characteristics of CFT titre, PCR load, MOMP-G IgG EPT and PmpG IgG EPT results of lambs that had recurring infections from 2 to 10 months of age.
(DOCX)

S3 Table Longitudinal data of individual animal tested in recurring infections with no CFT antibodies group. Characteristics of CFT titre, PCR load, MOMP-G IgG EPT and PmpG IgG EPT results of lambs that had recurring infections with no CFT antibodies detected from two to 10 months of age.
(DOCX)

S4 Table Cross-sectional data of individual animal tested in polyarthritis group. Characteristics of CFT titre, MOMP-G IgG and PmpG IgG EPT results of lambs with overt signs of polyarthritis.
(DOCX)

S5 Table Analyses of PmpG and MOMP specific epitope responses. Characteristics of epitope responses recorded in individual lambs and their corresponding groups of naturally infected lambs, lambs with polyarthritis and vaccine-induced lambs.
(XLSX)
