# Supplementary material for: Humoral immune response against two surface antigens of Chlamydia pecorum in vaccinated and naturally infected sheep
Source: PLoS One. 2017 Nov 30;12(11):e0188370. doi: 10.1371/journal.pone.0188370 (PMC5708773; doi:10.1371/journal.pone.0188370)
Supplement: S1 Table — Characteristics of CFT titre, PCR load, MOMP-G IgG EPT and PmpG IgG EPT results of lambs that cleared their infection by 10 month of age. (DOCX) [file pone.0188370.s001.docx]

| **Clearance of infection** | | | | | | | | | | | | | | |
| --- | --- | --- | --- | --- | --- | --- | --- | --- | --- | --- | --- | --- | --- | --- |
|  | **2 months** | | | |  | **6 months** | | | |  | **10 months** | | | |
| **Animal ID** | **CFT** | **PCR** | **MOMP-G** | **PmpG** |  | **CFT** | **PCR** | **MOMP-G** | **PmpG** |  | **CFT** | **PCR** | **MOMP-G** | **PmpG** |
| B42 | 8 | 0 | 0 | 0 |  | 128 | 30 | 5227 | 4268 |  | 8 | 0 | 3755 | 5512 |
| B7 | 8 | 0 | 1412 | 0 |  | 64 | 164 | 3469 | 4894 |  | 32 | 0 | 5034 | 4881 |
| OR40 | 8 | 0 | 0 | 0 |  | 64 | 13 | 5335 | 22494 |  | 8 | 0 | 0 | 3994 |
| OR38 | 8 | 0 | 2412 | 0 |  | 32 | 1048 | 52648 | 40956 |  | 8 | 0 | 43983 | 9728 |
| B46 | 8 | 0 | 0 | 0 |  | 32 | 163 | 1009 | 22319 |  | 8 | 0 | 2410 | 0 |
| B48 | 16 | 0 | 0 | 1116 |  | 128 | 4917 | 728 | 5957 |  | 8 | 0 | 2086 | 1969 |
| B50 | 8 | 0 | 0 | 0 |  | 64 | 74 | 8029 | 11731 |  | 64 | 0 | 2355 | 4177 |
| B8 | 8 | 0 | 0 | 0 |  | 32 | 3000 | 1842 | 2988 |  | 8 | 0 | 1594 | 1059 |
| B24 | 8 | 0 | 0 | 0 |  | 32 | 183 | 1024 | 24653 |  | 8 | 0 | 0 | 793 |
| OR37 | 8 | 0 | 0 | 0 |  | 32 | 18 | 4228 | 2540 |  | 128 | 0 | 84846 | 6096 |
| B25 | 8 | 0 | 0 | 0 |  | 128 | 5 | 10723 | 3520 |  | 64 | 0 | 12714 | 1353 |
| B10 | 8 | 0 | 6072 | 0 |  | 32 | 3884 | 5824 | 3757 |  | 8 | 0 | 2398 | 0 |
| B38 | 8 | 0 | 2870 | 0 |  | 32 | 10 | 8482 | 2780 |  | 8 | 0 | 13537 | 0 |
| No. of positives | 1 | - | 4 | 1 |  | 13 | 13 | 13 | 13 |  | 4 | - | 11 | 10 |
| % positive | 7.7 | - | 30.8 | 7.7 |  | 100 | 100 | 100 | 100 |  | 30.8 | - | 84.61 | 84.61 |

| **Recurring infections** | | | | | | | | | | | | | | |
| --- | --- | --- | --- | --- | --- | --- | --- | --- | --- | --- | --- | --- | --- | --- |
|  | **2 months** | | | |  | **6 months** | | | |  | **10 months** | | | |
| **Animal ID** | **CFT** | **PCR** | **MOMP-G** | **PmpG** |  | **CFT** | **PCR** | **MOMP-G** | **PmpG** |  | **CFT** | **PCR** | **MOMP-G** | **PmpG** |
| B57 | 8 | 0 | 1823 | 0 |  | 64 | 50 | 54321 | 1211 |  | 16 | 16 | 45428 | 3063 |
| B59 | 16 | 0 | 3976 | 0 |  | 32 | 70, 312^ | 32089 | 4031 |  | 8 | 15 | 12831 | 2639 |
| B85 | 8 | 0 | 0 | 1176 |  | 32 | 1000 | 3547 | 7372 |  | 8 | 11 | 1873 | 17676 |
| B79 | 8 | 0 | 6340 | 0 |  | 32 | 20 | 9977 | 2390 |  | 8 | 14 | 9691 | 7569 |
| B47 | 16 | 0 | 0 | 1162 |  | 8 | 0 | 5423 | 5831 |  | 64 | 1147 | 4024 | 15092 |
| B62 | 8 | 65 | 0 | 5774 |  | 16 | 0 | 0 | 5643 |  | 8 | 84 | 1189 | 13237 |
| Or90 | 8 | 31 | 0 | 2373 |  | 32 | 46 | 0 | 4707 |  | 8 | 1517 | 1923 | 28048 |
| No. of positives | 2 | 2 | 3 | 4 |  | 6 | 5 | 5 | 7 |  | 2 | 7 | 7 | 7 |
| % positive | 28.5 | 28.5 | 42.8 | 57.1 |  | 85.7 | 71.4 | 71.4 | 100 |  | 28.5 | 100 | 100 | 100 |

^PCR positive at more than one anatomical site

| **Recurring infections with no CFT antibodies** | | | | | | | | | | | |
| --- | --- | --- | --- | --- | --- | --- | --- | --- | --- | --- | --- |
| **Animal ID** | **Age** | **CFT** | **PCR** | **MOMP-G** | **PmpG** |  | **Age** | **CFT** | **PCR** | **MOMP-G** | **PmpG** |
| OR35 | 2m | 8 | 29 | 0 | 0 |  | 4m | 8 | 34, 1258^ | 0 | 11510 |
| B77 | 8m | 8 | 68, 281^ | 0 | 4902 |  | 10m | 8 | 36 | 2396 | 4684 |
| Y1575 | 6m | 8 | 9767 | 3402 | 19934 |  | 10m | 8 | 15 | 1433 | 9228 |

^PCR positive at more than one anatomical site

| **Polyarthritis** | | | | | |
| --- | --- | --- | --- | --- | --- |
| **Animal ID** | **CFT titre** | **MOMP-G EPT** | | **PmpG EPT** | |
| Ovine 4 | 128 | 14052 | | 6767 | |
| Ovine 7 | 128 | 15114 | | 3840 | |
| Ovine 8 | 128 | 86238 | | 2698 | |
| Ovine 35 | 128 | 12949 | | 2448 | |
| Ovine 37 | 128 | 29213 | | 45297 | |
| Ovine 51 | 128 | 42966 | | 4061 | |
| Ovine 56 | 128 | 2654 | | 3237 | |
| Ovine 5 | 64 | 12369 | | 850 | |
| Ovine 23 | 64 | 10705 | | 3235 | |
| Ovine 24 | 64 | 54959 | | 3664 | |
| Ovine 38 | 64 | 31674 | | 505 | |
| Ovine 53 | 64 | 18632 | | 300 | |
| Ovine 28 | 32 | 70326 | | 1952 | |
| Ovine 29 | 32 | 88083 | | 3215 | |
| Ovine 34 | 32 | 34782 | | 5231 | |
| Ovine 36 | 32 | 14024 | | 267 | |
| Ovine 39 | 32 | 4405 | | 12179 | |
| Ovine 52 | 32 | 34113 | | 11283 | |
| Ovine 54 | 32 | 22963 | | 27628 | |
| Ovine 25 | 32 | 1379 | | 15033 | |
| No. of positives | 20 | 20 |  | | 20 |
| % positive | 100 | 100 |  | | 100 |
