# Supplementary material for: Humoral immune response against two surface antigens of Chlamydia pecorum in vaccinated and naturally infected sheep
Source: PLoS One. 2017 Nov 30;12(11):e0188370. doi: 10.1371/journal.pone.0188370 (PMC5708773; doi:10.1371/journal.pone.0188370)
Supplement: S2 Table — Characteristics of CFT titre, PCR load, MOMP-G IgG EPT and PmpG IgG EPT results of lambs that had recurring infections from 2 to 10 months of age. (DOCX) [file pone.0188370.s002.docx]

| **Recurring infections** | | | | | | | | | | | | | | |
| --- | --- | --- | --- | --- | --- | --- | --- | --- | --- | --- | --- | --- | --- | --- |
|  | **2 months** | | | |  | **6 months** | | | |  | **10 months** | | | |
| **Animal ID** | **CFT** | **PCR** | **MOMP-G** | **PmpG** |  | **CFT** | **PCR** | **MOMP-G** | **PmpG** |  | **CFT** | **PCR** | **MOMP-G** | **PmpG** |
| B57 | 8 | 0 | 1823 | 0 |  | 64 | 50 | 54321 | 1211 |  | 16 | 16 | 45428 | 3063 |
| B59 | 16 | 0 | 3976 | 0 |  | 32 | 70, 312^ | 32089 | 4031 |  | 8 | 15 | 12831 | 2639 |
| B85 | 8 | 0 | 0 | 1176 |  | 32 | 1000 | 3547 | 7372 |  | 8 | 11 | 1873 | 17676 |
| B79 | 8 | 0 | 6340 | 0 |  | 32 | 20 | 9977 | 2390 |  | 8 | 14 | 9691 | 7569 |
| B47 | 16 | 0 | 0 | 1162 |  | 8 | 0 | 5423 | 5831 |  | 64 | 1147 | 4024 | 15092 |
| B62 | 8 | 65 | 0 | 5774 |  | 16 | 0 | 0 | 5643 |  | 8 | 84 | 1189 | 13237 |
| Or90 | 8 | 31 | 0 | 2373 |  | 32 | 46 | 0 | 4707 |  | 8 | 1517 | 1923 | 28048 |
| No. of positives | 2 | 2 | 3 | 4 |  | 6 | 5 | 5 | 7 |  | 2 | 7 | 7 | 7 |
| % positive | 28.5 | 28.5 | 42.8 | 57.1 |  | 85.7 | 71.4 | 71.4 | 100 |  | 28.5 | 100 | 100 | 100 |

^PCR positive at more than one anatomical site
