# Supplementary material for: Humoral immune response against two surface antigens of Chlamydia pecorum in vaccinated and naturally infected sheep
Source: PLoS One. 2017 Nov 30;12(11):e0188370. doi: 10.1371/journal.pone.0188370 (PMC5708773; doi:10.1371/journal.pone.0188370)
Supplement: S3 Table — Characteristics of CFT titre, PCR load, MOMP-G IgG EPT and PmpG IgG EPT results of lambs that had recurring infections with no CFT antibodies detected from two to 10 months of age. (DOCX) [file pone.0188370.s003.docx]

| **Recurring infections with no CFT antibodies** | | | | | | | | | | | |
| --- | --- | --- | --- | --- | --- | --- | --- | --- | --- | --- | --- |
| **Animal ID** | **Age** | **CFT** | **PCR** | **MOMP-G** | **PmpG** |  | **Age** | **CFT** | **PCR** | **MOMP-G** | **PmpG** |
| OR35 | 2m | 8 | 29 | 0 | 0 |  | 4m | 8 | 34, 1258^ | 0 | 11510 |
| B77 | 8m | 8 | 68, 281^ | 0 | 4902 |  | 10m | 8 | 36 | 2396 | 4684 |
| Y1575 | 6m | 8 | 9767 | 3402 | 19934 |  | 10m | 8 | 15 | 1433 | 9228 |

^PCR positive at more than one anatomical site
