# Supplementary material for: Humoral immune response against two surface antigens of Chlamydia pecorum in vaccinated and naturally infected sheep
Source: PLoS One. 2017 Nov 30;12(11):e0188370. doi: 10.1371/journal.pone.0188370 (PMC5708773; doi:10.1371/journal.pone.0188370)
Supplement: S4 Table — Characteristics of CFT titre, MOMP-G IgG and PmpG IgG EPT results of lambs with overt signs of polyarthritis. (DOCX) [file pone.0188370.s004.docx]

| **Polyarthritis** | | | | | |
| --- | --- | --- | --- | --- | --- |
| **Animal ID** | **CFT titre** | **MOMP-G EPT** | | **PmpG EPT** | |
| Ovine 4 | 128 | 14052 | | 6767 | |
| Ovine 7 | 128 | 15114 | | 3840 | |
| Ovine 8 | 128 | 86238 | | 2698 | |
| Ovine 35 | 128 | 12949 | | 2448 | |
| Ovine 37 | 128 | 29213 | | 45297 | |
| Ovine 51 | 128 | 42966 | | 4061 | |
| Ovine 56 | 128 | 2654 | | 3237 | |
| Ovine 5 | 64 | 12369 | | 850 | |
| Ovine 23 | 64 | 10705 | | 3235 | |
| Ovine 24 | 64 | 54959 | | 3664 | |
| Ovine 38 | 64 | 31674 | | 505 | |
| Ovine 53 | 64 | 18632 | | 300 | |
| Ovine 28 | 32 | 70326 | | 1952 | |
| Ovine 29 | 32 | 88083 | | 3215 | |
| Ovine 34 | 32 | 34782 | | 5231 | |
| Ovine 36 | 32 | 14024 | | 267 | |
| Ovine 39 | 32 | 4405 | | 12179 | |
| Ovine 52 | 32 | 34113 | | 11283 | |
| Ovine 54 | 32 | 22963 | | 27628 | |
| Ovine 25 | 32 | 1379 | | 15033 | |
| No. of positives | 20 | 20 |  | | 20 |
| % positive | 100 | 100 |  | | 100 |
